# Associations of lifetime stressors and health behaviors with inflammation in young adults previously placed in youth residential care

**DOI:** 10.1016/j.bbih.2025.101098

**Published:** 2025-09-10

**Authors:** David Bürgin, Kristen Nishimi, Vera Clemens, Maria Meier, Eva Unternaehrer, Laura Gurri, Evelyne Bruttin, Nicolas Rohleder, Paul Klauser, Daniella Dwir, Nimmy Varghese, Anne Eckert, Süheyla Seker, Delfine d’Huart, Cyril Boonmann, Marc Schmid, Aoife O'Donovan

**Affiliations:** a Child and Adolescent Psychiatric Research Department, University Psychiatric Hospitals, University of Basel, Basel, Switzerland; b Jacobs Center for Productive Youth Development, University of Zurich, Zurich, Switzerland; c Department of Psychiatry and Behavioral Sciences, University of California San Francisco, San Francisco, United States of America; d San Francisco Veterans Affairs Medical Center, San Francisco, CA, United States of America; e Department of Child and Adolescent Psychiatry/Psychotherapy, University Ulm, Ulm, Germany; f Department of Psychology, Division of Neuropsychology, University of Konstanz, Constance, Germany; g Department of Psychology, Chair of Health Psychology, Friedrich-Alexander-Universität Erlangen-Nürnberg, Germany; h Center for Psychiatric Neuroscience, Department of Psychiatry, Lausanne University Hospital and University of Lausanne, Lausanne, Switzerland; i Division of Child and Adolescent Psychiatry, Department of Psychiatry, Lausanne University Hospital and University of Lausanne, Lausanne, Switzerland; j Neurobiology Lab for Brain Aging and Mental Health, University Psychiatric Clinics Basel, Basel, 4002, Switzerland; k Research Cluster Molecular and Cognitive Neurosciences, University of Basel, 4002, Basel, Switzerland; l Department of Social Work, Stockholm University, Stockholm, Sweden; m LUMC Curium – Department of Child and Adolescent Psychiatry, Leiden University Medical Center, Leiden, the Netherlands

**Keywords:** Adversity, Maltreatment, Stressors, Inflammation, Health behaviors, Cytokines, Out-of-home care, Youth residential care, Care leaver

## Abstract

**Background:**

Early life stressors (ELS) and stressful life events (SLEs) increase the risk for various physical health conditions, and health behaviors can modulate stress-associated risks. A key mechanism linking both lifetime stress and health behaviors with physical health outcomes is chronic low-grade inflammation. However, it is unclear how both stressor exposure and more proximal health behaviors are associated with inflammation in highly stress-exposed groups.

**Objectives:**

Here, we investigated associations of lifetime stressors and health behaviors with peripheral inflammation in a highly stress exposed sample of young adults previously placed within youth residential care in Switzerland.

**Method:**

We examined 126 young adults (*M*_Age_ = 26.3 years; 31 % female) who completed questionnaires to assess ELS, SLEs, and risky and protective health behaviors. Inflammatory markers (C-reactive protein [CRP], interleukin [IL]-6, tumor necrosis factor [TNF]-α, IL-10, and IL-1ra) were measured in venous blood using high sensitivity enzyme-linked immunosorbent assays (hsELISAs). Regressions estimated associations between ELS, SLEs, and health behaviors with each inflammatory marker.

**Results:**

Our sample reported high levels of ELS and SLEs, as well as high levels of risky health behaviors. ELS and SLEs were mostly unassociated with young adult health behaviors, and both ELS and SLEs were not associated with inflammatory markers, adjusting for covariates. Regarding behavior, nicotine dependence was associated with higher pro-inflammatory markers and alcohol abuse marginally with a lower anti-inflammatory marker, while physical activity and better sleep quality were associated with lower pro-inflammatory markers, adjusting for covariates.

**Conclusions:**

Among individuals with high levels of lifetime stress, cumulative ELS and SLEs were unassociated with inflammation, whereas risky behaviors were associated with higher, and protective behaviors with lower inflammatory markers. Interventions that reduce risky and promote protective health behaviors may lower inflammation and promote long-term health among individuals who have experienced high lifetime stressors exposure.

## Highlights

• Stressors were not significantly associated with five inflammatory markers tested in young adults.
• Risky behaviors were related to higher pro- and lower anti-inflammatory markers.
• Protective behaviors were related to lower pro-inflammatory markers.
• Health behaviors were associated with inflammation in a highly stress exposed sample.

## 1 Introduction

Early life stressors (ELS) and stressful life events (SLEs) increase the risk for psychopathology, physical diseases, and premature mortality (Anda et al., 2006; Bellis et al., 2019; Brown et al., 2009; Dube et al., 2001; Felitti et al., 1998; Hughes et al., 2017; Kessler et al., 2010; Teicher et al., 2021). Multiple physiological systems, including the endocrine, immune, and redox-systems, play a role in the pathophysiological effects of stressor exposure (Nusslock and Miller, 2016; Slavich, 2020). Particularly strong evidence implicates dysregulated inflammation as a mechanism linking ELS and somatic morbidity (Baumeister et al., 2016; Coelho et al., 2014; Danese and Baldwin, 2017a; Lacey et al., 2020). Unhealthy patterns of behavior may contribute to heightened chronic inflammation among stressor-exposed individuals. Health behaviors are of particular interest and might be important targets for behavioral intervention in populations exposed to high levels of stressors. Yet, research investigating the effects of cumulative stressors and health behaviors among highly stress-exposed populations is sparse. This is concerning as we have a poor understanding of how cumulative stressors and health behaviors influence mechanisms of ill health in individuals who face a high burden of stressor exposure (McEwen and McEwen, 2017; Zelechoski et al., 2013). Therefore, we aimed to investigate associations of ELS, SLEs, and health behaviors with inflammatory markers in a high-risk population of young adults who were previously placed within youth residential care institutions in Switzerland.

Based on the conceptual framework of biological embedding and a lifecourse perspective, stressors occurring in early life are expected to impact health trajectories via physiological impacts on stress response systems (Berens et al., 2017; Knudsen, 2004; Ben-et al., 2016). Adverse experiences, particularly in sensitive periods of development like ELS, can contribute to dysregulated stress responses and ultimately heightened allostatic load, including chronic low-grade inflammation (Lupien et al., 2009; McEwen and Gianaros, 2010), which can in turn influence lifelong health. Indeed, exposure to ELS including childhood adversity, maltreatment, trauma, abuse, or neglect increases risk for adverse health outcomes across the entire lifetime (Bellis et al., 2019; Bürgin et al., 2021; McCrory et al., 2017; McLaughlin, 2016; McLaughlin et al., 2020). Compared to individuals without ELS, individuals with multiple stressor exposures in childhood have increased risks for internalizing mental health problems, obesity, diseases of aging (e.g., cardiovascular disease, diabetes, cancer, dementia), and premature mortality (Anda et al., 2006; Brown et al., 2009; Felitti et al., 1998; Kessler et al., 2010; Green et al., 2010; Heim and Binder, 2012; Lin et al., 2016; O'Donovan et al., 2012; Widom et al., 2007). ELS also increases the risk of individuals accumulating later SLEs throughout adulthood, which may further exacerbate risks for ill health (Bürgin et al., 2021; Ferraro and Shippee, 2009; Pearlin et al., 2005). Particularly, out-of-home placed children and adolescents are exposed to high rates of ELS (incl. child maltreatment such as abuse and neglect, trauma, or parental separation) (Zelechoski et al., 2013; Briggs et al., 2012; Greger et al., 2015; McLaughlin et al., 2015), usually being the main reason for out-of-home placement, or a consequence of living in the care system. In sum, ELS can have lasting negative sequelae and out-of-home placed children and adolescents are prone to these long-lasting effects due to their high exposure to both ELS and SLEs.

Inflammation is a key aspect of allostatic load and mechanism linking ELS and SLEs with long-term adverse health outcomes (Slavich, 2020; Danese and Baldwin, 2017a). Inflammatory activity, in particular the release of inflammatory cytokines, is a central innate immune response to anticipated and actual threat exposure, physical trauma, and infection, and is a highly adaptive acute response that protects humans from harm and enhances survival (Slavich, 2020; Danese and Baldwin, 2017b; O'Donovan et al., 2013). However, prolonged activation of this system – for instance due to persistent objective or perceived threat exposures or increased threat sensitivity – can be damaging in the long run and may be a key physiological pathway linking ELS with psychopathology and chronic disease (O'Donovan et al., 2013; Baldwin and Danese, 2019; Furman et al., 2019). A recent meta-analysis summarizing 27 studies found moderate associations between ELS and inflammatory markers among children and adolescents, although there was significant variability across study methodologies (Kuhlman et al., 2020). Another meta-analysis of 25 adult studies reported small associations between childhood trauma and inflammation, with findings varying by trauma type (Baumeister et al., 2016). In addition to markers broadly considered “pro-inflammatory”, (C-reactive protein (CRP), interleukin-6 (IL-6), and tumor necrosis factor-alpha (TNF-α) (Baumeister et al., 2016; Kuhlman et al., 2020)), other studies have included “anti-inflammatory” markers, such as IL-10 and IL-1 receptor antagonist (IL-1ra) which are generally immunoregulatory with net anti-inflammatory effects (Corsi-Zuelli et al., 2020; Goldstein et al., 2021; Heard-et al., 2020; Marsland et al., 2017). Taken together, meta-analyses suggest that stressor exposure is associated with elevated inflammation; however, there is considerable heterogeneity across studies, and few have incorporated anti-inflammatory markers (Baumeister et al., 2016; Kuhlman et al., 2020).

Health behaviors play a significant role in determining overall mental and physical well-being. These behaviors encompass various actions with implications for physical health, commonly classified into two categories: risky and protective (Glanz et al., 2008; Lee and Park, 2018; Nishimi et al., 2022). Research on stress and trauma has predominantly focused on risky behaviors, including smoking, alcohol consumption, and drug use, while protective health behaviors like healthy eating and physical activity have received less attention (Lee and Park, 2018; Rheingold et al., 2004). Both risky and protective health behaviors tend to cluster together within individuals (e.g., one might show high levels of multiple risky behaviors), suggesting that these behaviors have common underpinnings and may influence each other (Poortinga, 2007; Schuit et al., 2002). However, there is some evidence of mixed behavioral patterns (e.g., high levels of both risky and protective behavior) (Nishimi et al., 2022), thus it is critical to examine both dimensions separately and concurrently. Health behaviors are related to lifetime stressor exposure (Duffy et al., 2018), with individuals exposed to ELS tending to engage in more risky and fewer protective health behaviors (Duffy et al., 2018). Risky behaviors such as smoking, alcohol consumption, and sleep disruption increase inflammation, while protective behaviors like healthy diet and exercise can reduce inflammation (O'Connor and Irwin, 2010). Considering biological embedding of ELS and lifecourse timing, we may hypothesize that behavior patterns in young adulthood could buffer or potentiate the impact of previous stressors on inflammation (Raposa et al., 2014). For example, despite high stressor exposure, fewer risky and more protective behaviors may confer reduced inflammation and better health (Puterman et al., 2010). Thus, consideration of health behaviors is needed for a comprehensive understanding of the relationship between lifetime stressor exposure and inflammation.

In the current study, we investigated whether both lifetime stressors and health behaviors were associated with pro- and anti-inflammatory markers in a sample of previously out-of-home placed young adults. Prior research indicates that this sample showed very high rates of ELS exposure shown to be associated with higher psychopathology and poorer psychosocial functioning (Schmid et al., 2022; Bürgin et al., 2025; Meier et al., 2024), as well as with dysregulated stress-systems (e.g., longer telomere length, lower hair cortisol concentrations, and epigenetic age deceleration) (Meier et al., 2024; Bürgin et al., 2022). Thus, such populations are at higher risk for various types of stressors and associated health risks, however, it is unclear if health behaviors are promising potential treatment targets in this group. To remedy this, we examined association of ELS and SLEs, as well as risky and protective health behaviors with peripheral inflammatory markers (i.e., CRP, IL-6, TNF-α, IL-10, and IL-1ra) in a high-risk sample of young adults previously placed within youth residential care institutions in Switzerland. We hypothesized that experiences of ELS and SLEs, and risky health behaviors would be associated with higher pro-inflammatory/lower anti-inflammatory activity, while protective health behaviors would be associated with lower pro-inflammatory/higher anti-inflammatory activity. While ELS is often associated with increased risk for later SLEs (Bürgin et al., 2021; Ferraro and Shippee, 2009; Pearlin et al., 2005), based on lifecourse models, we might anticipate early compared to lifetime or more recent experiences having distinct impacts on inflammation, thus we estimated their independent effects (e.g., co-adjusted for each other). Relatedly, the patterns of both risky and protective behaviors together are relevant, so co-adjusted associations of behaviors with inflammation were estimated. In exploratory analyses, we estimated the moderation between ELS/SLEs and health behaviors to determine whether any lifetime stressor-inflammation associations were influenced by more proximal health behaviors.

## 2 Methods

### 2.1 Study procedures and design

We used data from a longitudinal cohort study of young people in youth residential care. Initially, youths were recruited to participate in the cohort study “Swiss Study for Clarification and Goal-Attainment in Child Welfare and Juvenile-Justice Institutions” (German: “Modellversuch Abklarung und Zielerreichung in stationaren Massnahmen”; MAZ.), when they were placed in a Swiss residential youth care institution between the years 2007–2011 (Schmid et al., 2022; Jäggi et al., 2021). The MAZ. study had the primary aim of examining mental health and behavior of children, adolescents, and young adults in residential care institutions. The included institutions were all accredited by the Swiss Federal Ministry of Justice and represent the various types of Swiss youth institutions with regards to size, schooling opportunities, treatment options, and specific placements of children and adolescents. Participants were placed due to civil law, penal law, or voluntarily. Participants with non-sufficient language skills in either German, French or Italian were excluded from the MAZ. sample (Schmid et al., 2022).

A total of 231 MAZ. participants were reassessed as part of a longitudinal 10-year follow-up “Youth Welfare Trajectories: Learning from Experience” (JAEL) study between the years 2018–2020. Participants completed online questionnaires and a face-to-face assessment with the study team, using structured clinical interviews. The JAEL study aimed to investigate the psychosocial health of young adults with previous residential care placements (Schmid et al., 2022, d’Huart et al., 2022; Kind et al., 2023; Seker et al., 2022a). In a biomarker add-on study, biological specimens were collected for biomarker assays. Participants with a current common cold/flu or current or chronic known immunological diseases (e.g., HIV) were excluded from biological sampling. All participants signed an informed consent for participation in the longitudinal follow-up study and additionally for the biomarker add-on. A figure displaying participant flow leading to the final sample size of 126 is provided in the supplementary material (Suppl Fig. 1). Attrition analyses showed a slight overrepresentation of people with Swiss nationality in the follow-up study, but there were no other significant differences between included and non-included participants regarding sociodemographic or psychosocial factors (Suppl Table 1), nor criminal records over time (through December 2017; Suppl Table 2). All data for the current analyses were collected from the JAEL follow-up study, besides sex which was assessed in the baseline MAZ. study. The Ethics Commission of Northwestern Switzerland reviewed and approved the follow-up study design and procedures, including its biomarker add-on study (EKNZ, Ref. 2017-00718).

### 2.2 Measures

#### 2.2.1 Lifetime stressors

##### 2.2.1.1 Early life stressors (ELS)

Early life stressors (ELS) was assessed using the “Maltreatment and Abuse Chronology of Exposure (MACE)” scale (Teicher and Parigger, 2015). The 75-item version of the MACE-X was administered during face-to-face assessments as self-filled out questionnaire on a tablet (Isele et al., 2014). The questionnaire assesses severity of exposure to ten different types of stressors during each year from birth to age 18 years, including different forms of abuse and neglect by parents, emotional and physical violence by peers, and witnessing violence towards a parent or sibling (Isele et al., 2014). The MACE provides an overall score for both severity and multiplicity (number of types of adversities) and shows excellent test-retest reliability (Teicher and Parigger, 2015). We used the MACE multiplicity score as a surrogate for the degree of ELS exposure, which aligns with the operationalization of stressful life events in this study as well as commonly used adverse childhood experiences scale elsewhere (Petruccelli et al., 2019).

##### 2.2.1.2 Stressful life events (SLEs)

Stressful life events (SLEs) were measured with the “Life Events Checklist-Revised” (LEC-R) (Gray et al., 2004). The LEC-R assesses the experience of stressful and potentially traumatizing life events (e.g., accident or natural disaster, physical assault, life-threating injury or disease, death of a loved one), at any point in the lifetime (Gray et al., 2004). The scale includes 19 stressful events that participants can indicate as having ever experienced, witnessed or heard about. It was administered as an online screening tool. All events that were directly experienced or witnessed were summed to derive a cumulative SLE score.

#### 2.2.2 Health behaviors

##### 2.2.2.1 Risky health behaviors

***Nicotine dependence*** was assessed with the Fagerström Test for Nicotine Dependence (FTND) (Fagerström, 1978). The FTND is a self-report measure of six questions derived as a total score that reflects current nicotine dependence (five categories from none to very severe dependence). We dichotomized the final score and coded any dependence as current nicotine dependence. ***Cannabis dependence*** was assessed with the Severity of Dependence Scale (SDS) Cannabis subscale (Gossop et al., 1995). Next to determining the frequency of consumption, the five-item questionnaire includes the following questions: (1) Did you think your use was out of control? (2) Did the prospect of missing a dose make you anxious/worried? (3) Did you worry about your substance use? (4) Did you wish you could stop using? (5) How difficult would you find it to stop or go without the substance use? Items are scored on a 4-point scale; 0 = never/not difficult; 1 = sometimes/quite difficult; 2 = often/very difficult; 3 = always/impossible. We dichotomized the score using the established cut-off (≥4) to indicate screening positive for cannabis dependence (Kind et al., 2023). ***Risky alcohol use*** was measured with the Alcohol Use Disorders Identification Test (AUDIT) (Saunders et al., 1993). The AUDIT is a well-established multilingual instrument to screen for hazardous and harmful alcohol consumption (Saunders et al., 1993), and has been validated with multinational populations of different ages (Berner et al., 2007; Kuitunen et al., 2018). The 10-item questionnaire assesses alcohol consumption, drinking behavior, and alcohol-related problems with items rated on a 5-point frequency scale (0 = never to 4 = daily or almost daily). We summed the items to obtain a total score, and a cut-off (≥8) was used to define current risky alcohol use (Saunders et al., 1993).

##### 2.2.2.2 Protective health behaviors

***Nutrition*** was assessed with a screener consisting of five binary items asking if participants consumed whole foods, fruit and vegetables, or food containing omega-3 on more than three days, and consumed processed meat or sweetened beverages less than three times per week (Blackburn and Epel, 2017). We created a total score ranging from 0 to 5, with higher scores indicating healthier nutritional habits. ***Physical activity*** in the last month was measured on a single 6-point scale (1 = not much physical activity to 6 = almost daily [≥5 days per week] intensive physical activity) using the Stanford leisure-time activity categorical item (L-CAT) (Kiernan et al., 2013). ***Sleep quality*** were assessed using the ‘Pittsburgh Sleep Quality Index’ (PSQI) (Buysse et al., 1989). The questionnaire consists of different response formats assessing subjective experiences and objective parameters of sleep. We summed up all nine items assessing sleep disturbances scores (Range 0–27; higher scores indicating poorer sleep quality) (Buysse et al., 1989; Varghese et al., 2023). For interpretive purposes, we reverse coded total sleep disturbance scores such that higher scores indicate better sleep quality.

#### 2.2.3 Inflammatory markers

Blood samples were collected in the morning of the face-to-face assessment between 8.30 a.m. and 11.00 a.m. (11.9 % after 11 a.m.) between January 2018 and July 2020. Serum clot activator tubes (CAT) and ethylenediaminetetraacetic acid (EDTA) plasma tubes (Becton Dickinson, BD) were centrifuged at 3000 g for 10 min. Aliquoted samples were subsequently stored at −80 °C before being shipped to the laboratory on dry ice for analysis. ***C-reactive protein (CRP)*** measurement was performed in serum using a commercially available hsELISA kit (DRG International, Inc., USA). Intra- and inter-assay coefficients of variation (CV) were 5.5 % and 11.3 %, respectively. ***Interleukin-6 (IL-6)*** measurement was performed in plasma using a hsELISA kit (R&D Systems, Inc., Minneapolis, MN, USA) with a lower detection limit of 0.09 pg/mL and an upper detection limit of 10 pg/mL. Intra- and inter-assay CVs were 6.7 %, and 6.1 %, respectively. Two participants (1.6 %) had IL-6 levels below and one participant (0.8 %) had levels above the detection limit; we imputed with corresponding values of the lower and upper detection limits. ***Tumor necrosis factor-alpha (TNF-α)*** measurement was performed in plasma using a hsELISA kit (R&D Systems, Inc., USA). Intra- and inter-assay CVs were 5.2 %, and 7.7 %, respectively. The lower detection limit was 0.049 pg/mL. One participant had TNF-*α* levels below the detection limit; thus the lower detection limit value was imputed*.* ***Interleukin-10 (IL-10)*** was measured in plasma using a hsELISA kit (R&D Systems, Inc., USA). No intra- and inter-assay CVs are available, as the sample amount was only sufficient for single measurement. The lower detection limit was 0.17 pg/mL; 23 participants (18.2 %) were below the detection limit, and the lower detection limit value was imputed. ***Interleukin-1 receptor antagonist (IL-1ra)*** measurement was performed in plasma using a hsELISA kit (R&D Systems, Inc., USA). Intra- and inter-assay CVs were 5.4 % and 2.4 %, respectively. The lower detection limit was 18.3 pg/mL and upper detection limit 2000 pg/mL. All participants had values within the detection limits.

#### 2.2.4 Covariates

Covariates included age, sex assigned at birth coded from institutional records (0 = male, 1 = female), and body mass index (BMI; in kg/m^2^).

### 2.3 Analytical plan

All participants with complete data on all five inflammatory markers (N = 126) were included in the analyses. Descriptive statistics and exploratory Spearman's correlational analyses were conducted (Table 1 and Suppl Table 3). Descriptive analyses showed four inflammatory markers to be skewed (CRP, IL-6, IL-10, IL-1ra), these markers were log-transformed to approximate normal distribution, all markers were then standardized (z-scaled; see Suppl Table 4). Patterns of missingness were investigated; 28.6 % of participants had at least one missing value in one of the study variables (most missing values were BMI). We imputed missing data for regression models using multivariate imputation by chained equation (k = 20 imputations) with its implementation in the ‘MICE’ package using predictive mean matching (PMM) (Buuren and Groothuis-Oudshoorn, 2011). Estimates from models in imputed datasets were then pooled using Rubin's rules to account for both within- and between-imputation variability (Buuren and Groothuis-Oudshoorn, 2011). Analyses were conducted with R Version 4.2.2 through RStudio version 2024.12.0 + 467 (Posit, Boston, MA, USA) (R Core Team, 2022).

**Table 1:** Descriptive information of covariates, early life stress (ELS), stressful life events (SLEs), and risky and protective health behaviors (*N* = 126).

	M or N	SD or %	Range
Covariates			
Age (years)	26.32	3.56	[16.1, 38.7]
Sex (Female)	39	31.0	
Number of Placements	3.87	3.94	[1, 20]
BMI (*n* = 98)	24.83	4.96	[16.1, 39.1]
**Lifetime Stressors**			
ELS (MACE Multiplicity)	3.21	2.38	[0, 10]
ELS (MACE Multiplicity, ≥1 scale above cutoff)	113	89.7	
SLEs (LEC-R)	4.64	2.79	[0, 12]
**Health Behaviors**			
Nicotine Dependence	69	54.8	
Cannabis Dependence	26	20.8	
Risky Alcohol Use (*n* = 125)	39	31.2	
Physical Activity	3.13	1.50	[1, 6]
Nutrition	2.24	1.28	[0, 5]
Sleep Quality	14.72	4.30	[0, 27]

Notes. BMI = body mass index, ELS = early life stress, LEC-R = Life Events Checklist-Revised, M = Mean, MACE = Maltreatment and Abuse Chronology of Exposure scale, SD = Standard Deviation, SLE = stressful life events.

We conducted sets of linear regression models with a) lifetime stressors (ELS and SLEs) and b) health behaviors (separate indicators for each risky and protective behavior) predicting each inflammatory marker separately, adjusting for covariates (age, sex, and BMI). A set of models then co-adjusted for lifetime stressors and health behaviors predicting each inflammatory marker, adjusting for covariates. All continuous variables were entered standardized, all binary variables were coded 0 or 1 (0 = feature absent, and 1 = feature present). Exploratory models examined interactions between lifetime stressor variables ELS and SLEs with each other, as well as ELS and SLEs separately and all health behavior variables, adjusting for covariates. We ran sensitivity analyses refitting models while excluding observations with CRP levels higher than 10 mg/L (*n* = 6), which can indicate acute inflammation, but may also reflect chronic inflammation (Ishii et al., 2012). Model assumptions were checked for all models on the first of the imputed datasets. Correlation analyses, pooling and visualization of parameter from regression models, and checking of model assumptions were performed using the ‘easystats’ ecosystem, including the packages ‘parameters’, ‘see’, ‘performance’, and ‘correlation’ (Lüdecke et al., 2020, 2021; Makowski et al., 2020).

## 3 Results

### 3.1 Descriptives

Participants had a mean age of 26.3 years (*SD* = 3.6; Range [16.1; 38.7]), and were 69 % male, and 90.9 % Swiss nationality. All participants were placed within Swiss youth residential care institutions at least once while growing up, however, most had numerous different placements in different institutions (mean number of different placements = 3.9, *SD* = 3.9; Range [1, 20]). About half of all participants were placed based on a civil law jurisdiction (*n* = 62; 52.1 %), about a quarter each based on a penal law jurisdiction (n = 29; 24.4 %) and other reasons (*n* = 28; 23.5 %) at the time when entering the cohort study. Most of the sample reported at least one form of ELS (89.7 % above cut-off on at least one of the ten MACE subscales), with many reporting multiple forms of ELS (*M*_scales above cut-off_ = 3.2, *SD* = 2.4, Range [0, 10]). Additionally, participants reported a mean of 4.6 self-experienced or witnessed SLEs (*SD* = 2.8; Range [0, 12]; Table 1).

Regarding risky health behaviors, 54.8 % of participants screened positive for nicotine dependence, 20.8 % participants screened positive for cannabis dependence, and 31.2 % screened positive for risky alcohol use (Table 1). Protective behaviors varied, with participants showing a moderate level of physical activity and moderately healthy nutrition (Table 1). While more than half of participants had a normal BMI (58.2 %; BMI = [18.5, 24.9]), about a quarter was overweight (25.5 %; BMI = [25, 29.9]), more than a tenth was obese (13.3 %; BMI>30), and only a few participants were underweight (3.1 %; BMI<18.5). Overall, the sample was characterized by numerous life stressors, high levels of risky health behaviors, and moderate levels of protective health-behaviors.

We observed a few significant bivariate Spearman correlations between ELS, SLEs, and health behaviors with inflammatory markers (Suppl Table 3). Risky and protective behaviors were modestly intercorrelated (i.e., risky behaviors *r*s = 0.17 to 0.19; protective behaviors *r*s = 0.01 to 0.27), and risky behaviors tended to be negatively correlated or non-correlated with protective behaviors (*r*s = −0.28 to 0.01). Additionally, higher ELS was associated with higher IL-1ra (*r* = 0.19), nicotine dependence was positively associated with higher CRP (*r* = 0.30) and IL-6 (*r* = 0.36), and physical activity (*r* = −0.18) and nutrition (*r* = −0.20) were negatively correlated with IL-6 (all *r*s p < 0.05).

### 3.2 Association of ELS, SLEs, and health behaviors with inflammatory markers

**Fig. 1:**
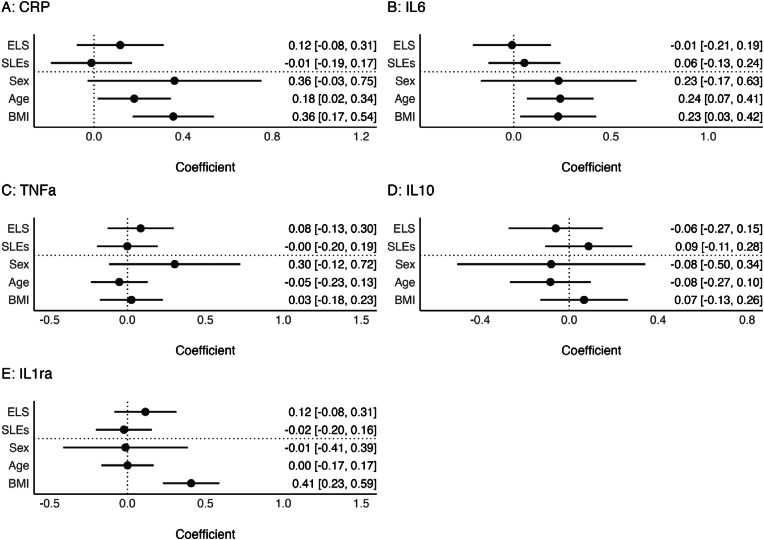
Associations of ELS and SLEs with inflammatory markers. **Notes.** Pooled estimates of multiple regression models across all imputed datasets. Different inflammatory markers are predicted by early life stressors (ELS), stressful life events (SLEs), age, sex, and BMI. All dimensional variables are standardized, all binary variables are coded using zero and one. Error bars represent pooled 95 %-Confidence Intervals. Within the supplementary materials tables of these regression models are provided in Supplementary Table 5.

**Fig. 2:**
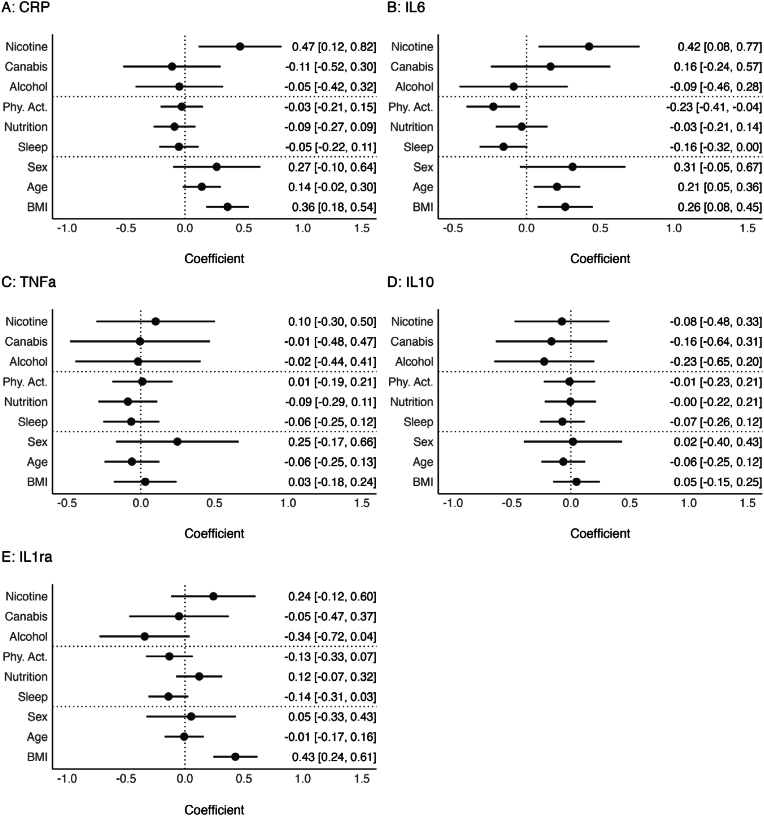
Associations of risky and protective health behaviors with inflammatory markers. **Notes.** Pooled estimates of multiple regression models across all imputed datasets. Different inflammatory markers are predicted by individual risky and protective health behaviors, age, sex, and BMI. All dimensional variables are standardized, all binary variables are coded using zero and one. Error bars represent pooled 95 %-Confidence Intervals. Within the supplementary materials tables of these regression models are provided in Supplementary Table 6.

**Fig. 3:**
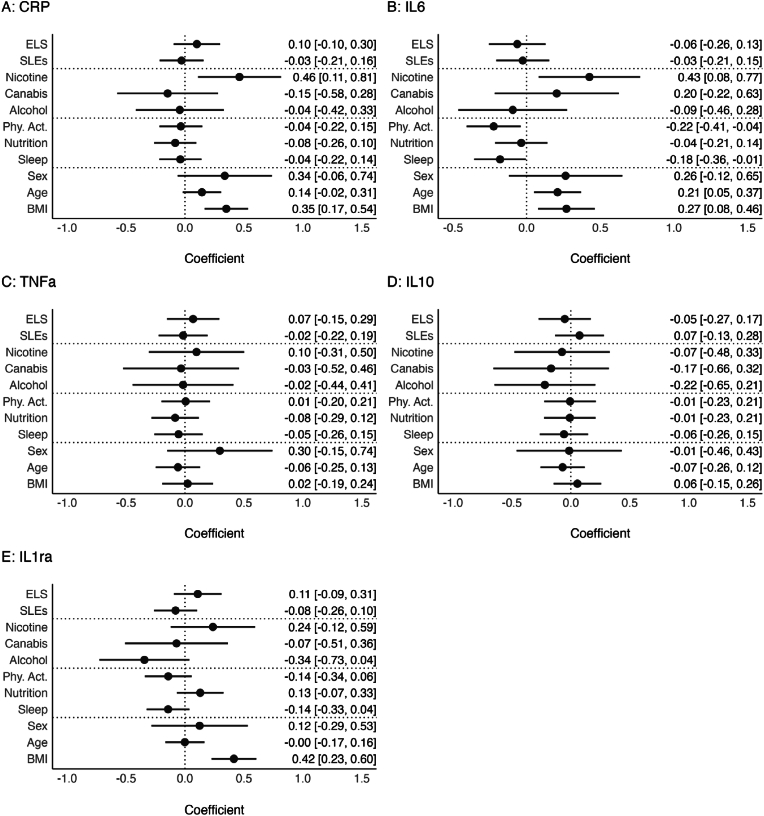
Associations of ELS, SLEs, and risky and protective health behaviors with inflammatory markers. **Notes.** Pooled estimates of multiple regression models across all imputed datasets. Different inflammatory markers are predicted by ELS, SLEs, individual risky and protective health behaviors, age, sex, and BMI. All dimensional variables are standardized, all binary variables are coded using zero and one. Error bars represent pooled 95 %-Confidence Intervals. Within the supplementary materials tables of these regression models are provided in Supplementary Table 7.

We fit separate linear regression models to predict CRP, IL-6, TNF-α, IL-10, and IL-1ra by a) ELS and SLEs, and b) risky and protective health behaviors, controlling for age, sex, and BMI (Fig. 1, Fig. 2 and Suppl Tables 5-6). First, we found no significant association of ELS and SLEs (when co-adjusting for each other) with any inflammatory marker in covariate adjusted models (Fig. 1 and Suppl Table 5); there were also no significant interactions between ELS and SLEs (see Suppl Table 5). Second, regarding risky behaviors, nicotine dependence was associated with significantly higher levels of CRP and IL-6, and risky alcohol use was marginally associated with lower IL-1ra (p = 0.08; Fig. 2 and Suppl Table 6). Regarding protective behaviors, physical activity and sleep (marginal, p = 0.06) were associated with lower IL-6 (Fig. 2 and Suppl Table 6). Third, associations were largely similar when co-adjusting ELS, SLEs, and health behaviors (Fig. 3 and Suppl Table 7). There were no significant predictors of TNF-α or IL-10 across models, and variance explained was low (model R^2^s 0.02–0.04).

### 3.3 Exploratory and sensitivity analyses

Exploratory models estimating ELS and SLEs interactions with individual health behaviors mostly indicated no significant effect modification. However, for CRP and TNF-α, the ELS∗nutrition interactions suggested that nutrition is associated with lower inflammation only among those with higher (ELS∗nutrition for CRP β = −0.17, 95 %CI -0.36, 0.01; for TNF-α β = −0.25, 95 %CI -0.46, −0.04), but not lower ELS.

Patterns identified in the primary models were equally apparent, if not slightly stronger, in sensitivity analyses excluding all participants with CRP-value of higher than 10 mg/L (*n* = 6).

## 4 Discussion

To our knowledge, this is the first study investigating multiple inflammatory biomarkers in previously out-of-home placed young adults, an understudied population at risk for stress-related pathology later in life. Among young adults previously placed in out-of-home care, we observed high cumulative lifespan stress exposure, in combination with high levels of risky and moderate levels of protective health behaviors. Notably, while risky and protective behaviors tended to co-occur among themselves (e.g., higher nicotine use and higher cannabis use), while being negatively correlated to one another (e.g., higher risk and lower protective behaviors), all correlations were only modest indicating varying patterns across different risk and protective behaviors (Nishimi et al., 2022). ELS and SLEs were not significantly associated with inflammatory markers, both in confounder-adjusted models and while adjusting for proximal health behaviors. In contrast, risky behavior engagement was associated with elevated pro-inflammatory markers and lower anti-inflammatory markers, adjusting for lifetime stress. Additionally, protective behaviors were associated with lower pro-inflammatory markers. These findings highlight the need to assess both risky and protective behaviors as well as pro- and anti-inflammatory markers to understand complex psychobiological relationships.

Lifetime stressors measured in young adulthood were largely unassociated with inflammatory markers in this high-risk sample. When estimating independent associations of ELS and SLEs in the same model (correlation of *r* = 0.33), no association between either lifetime stress measure and pro- or anti-inflammatory markers remained significant after controlling for health behaviors and covariates. This is not fully consistent with prior research in the field, which suggests small but significant associations between stressor exposure and higher inflammation on average (Baumeister et al., 2016; Kuhlman et al., 2020). However, our entire sample had experienced at least one out-of-home placement, oftentimes considered a stressful life event in itself. Young adults previously placed in youth residential care accumulate a broad array of risk factors across the lifetime in the context of limited socio-ecological resources (Schmid et al., 2022; Jäggi et al., 2021; Kind et al., 2023; Seker et al., 2022b; Baldwin et al., 2023). This population is also prone to selective attrition and healthy survivor effects, leading to more dropout rates over time due to selective morbidity or mortality, imprisonment, or institutionalization among highly burdened individuals (Bürgin et al., 2020; Küffer et al., 2016). Moreover, despite the relatively young age of the participants in the present study, average CRP levels in the sample were elevated (median = 1.73 mg/L while >1 mg/L is considered moderately increased) (Ridker and Cook, 2004). Therefore, the current sample represents individuals with high levels of stressors and elevated inflammation. Our findings, thus, indicate that relative increases in stressor exposure per se may not significantly impact inflammation at the upper end of the stress and adversity continuum. Further research is needed in other high-exposure groups, with larger samples and repeated longitudinal assessments that incorporate psychosocial, behavioral, and biological factors to extend our current findings.

Risky and protective health behaviors were associated with pro- and anti-inflammatory markers, with patterns in expected directions. In our high-risk sample, we found that nicotine dependence was associated with higher levels of pro-inflammatory markers CRP and IL-6, while alcohol use was associated with lower levels of anti-inflammatory marker IL-1ra. Notably, IL-1ra is considered immunoregulatory due to its action of blocking IL-1α and IL-1β pro-inflammatory cytokine signaling, thereby limiting excessive inflammatory response and preventing tissue damage (Abbate et al., 2020). However, IL-1ra levels may suggest higher levels of IL-1α and/or IL-1β, givens its production increases to provide negative feedback. As we could not measure IL-1 cytokines and did not have repeated measures to better assess immune activation dynamics, interpretation of this marginal association with alcohol use should be made cautiously. Higher levels of physical activity and sleep quality were associated with lower levels of IL-6. Other behaviors (i.e., cannabis dependence, nutrition) were not significantly associated with inflammatory markers. These results are generally consistent with prior work showing positive relationships between tobacco and alcohol use and pro-inflammatory markers, and negative relationships between regular physical activity and lower pro-inflammatory markers (O'Connor and Irwin, 2010). Our findings underscore the importance of assessing both risky and protective behavior dimensions, as both the reduction of risky health behaviors (Kind et al., 2023), and the promotion of beneficial health behaviors are promising behavioral intervention targets in high-risk populations. Additionally, our results indicate different patterns of associations depending on the specific inflammatory marker, indicating the need to consider both pro- and anti-inflammatory markers concurrently to gain a more nuanced characterization of inflammation. In contrast with our findings regarding stressor exposure, health behaviors do appear to impact inflammation at the higher end of the stress and adversity continuum.

When considering both stressors and health behaviors in analytical models simultaneously, estimates were largely unaffected and very few significant interactions were identified. This suggests that, despite some modest correlations, there was little covariance between stressors and behaviors in their associations with inflammatory markers, and that associations between behaviors and inflammation largely did not depend on the level of reported lifetime stressor exposure. However, exploratory findings suggested that associations between ELS and both CRP and TNF-α were influenced by nutrition status, such that among those with high ELS, better nutrition was associated with lower inflammation. Overall, these co-adjusted and moderation models indicated that proximal risky and protective behaviors in young adulthood were more strongly related to measures of circulating inflammation than lifetime stressors within our high-risk sample.

### 4.1 Limitations

Our findings should be interpreted considering some limitations. First, data are cross-sectional. Thus, despite retrospective reporting of past exposures and current health behaviors, directionality or causality cannot be determined. Second, we utilized retrospective measures of ELS and SLEs, which may be subject to biases such as recall biases (Bürgin et al., 2020; Baldwin et al., 2019; Danese, 2020). Third, no control group of young people without residential care placements was assessed, thus, our findings indicate relationships only within this young, high-risk, highly selective sample, and additional work comparing this sample with stress-unexposed groups could better contextualize our findings. Of note, given the young age of participants, chronic inflammation and its potential health manifestations may not yet be evident; it is possible that different patterns of associations between stressors, behaviors, and inflammation could emerge later in the lifecourse (Brody et al., 2023; Bahorik et al., 2024). Fourth, we lacked data on covariates which could have related to both our exposures and outcomes, such as ethnicity (Schmeer and Tarrence, 2018). Fifth, we may have been underpowered to detect some true small effects (particularly effect modification). Thus, we did not adjust models for out-of-home placement characteristics, such as cumulative time or the number of different types of institutions, as our sample size was insufficient. Lastly, we included BMI as a covariate given its relationship with inflammation (Heredia et al., 2012), though it could have been considered with health behaviors. BMI is a widely used anthropometric index, which is influenced by and related to multiple genetic, psychological, behavioral, and physical dimensions (Cameron et al., 2015). We acknowledge that BMI may also have complex relationships to stressor experience and health behaviors. Despite these shortcomings, our research adds important findings on the impacts of cumulative adverse exposures and later health behaviors on inflammation in a rare sample of young adults with previous residential care placements. Further investigations and replications in large samples are warranted to build upon these initial findings.

### 4.2 Conclusion

Our study is the first to present data on inflammatory markers in young adults who were previously placed in residential youth care in Switzerland. We observed a high prevalence of ELS and SLEs and high levels of risky health behaviors, and only modest levels of protective behaviors. Some risky and protective health behaviors, but not ELS and SLEs, were associated with inflammatory markers. High risk groups, such as young adults with previous residential out-of-home care, may benefit from behavioral interventions to bolster healthy behavior patterns, which in turn may result in lower inflammation. In sum, to improve the well-being of high-risk populations, it is crucial to develop prevention and intervention strategies that can effectively reduce exposure to stressors while simultaneously decreasing risky and promoting protective health behaviors.

## CRediT authorship contribution statement

**David Bürgin:** Conceptualization, Data curation, Formal analysis, Funding acquisition, Investigation, Methodology, Project administration, Validation, Visualization, Writing – original draft. **Kristen Nishimi:** Formal analysis, Investigation, Methodology, Validation, Writing – original draft, Writing – review & editing. **Vera Clemens:** Conceptualization, Funding acquisition, Investigation, Methodology, Supervision, Validation, Writing – review & editing. **Maria Meier:** Conceptualization, Investigation, Methodology, Validation, Writing – review & editing. **Eva Unternaehrer:** Funding acquisition, Investigation, Methodology, Validation, Writing – review & editing. **Laura Gurri:** Investigation, Methodology, Validation, Writing – review & editing. **Evelyne Bruttin:** Data curation, Formal analysis, Investigation, Methodology, Writing – review & editing. **Nicolas Rohleder:** Data curation, Investigation, Methodology, Project administration, Supervision, Validation, Writing – review & editing. **Paul Klauser:** Conceptualization, Funding acquisition, Investigation, Project administration, Resources, Writing – review & editing. **Daniella Dwir:** Conceptualization, Funding acquisition, Investigation, Methodology, Validation, Writing – review & editing. **Nimmy Varghese:** Data curation, Investigation, Methodology, Project administration, Writing – review & editing. **Anne Eckert:** Conceptualization, Data curation, Resources, Supervision, Validation, Writing – review & editing. **Süheyla Seker:** Data curation, Investigation, Validation, Writing – review & editing. **Delfine d’Huart:** Data curation, Investigation, Validation, Writing – review & editing. **Cyril Boonmann:** Conceptualization, Data curation, Investigation, Project administration, Supervision, Validation, Writing – review & editing. **Marc Schmid:** Conceptualization, Funding acquisition, Investigation, Project administration, Resources, Supervision, Validation, Writing – review & editing. **Aoife O'Donovan:** Conceptualization, Funding acquisition, Investigation, Methodology, Supervision, Validation, Writing – original draft, Writing – review & editing.

## Funding

This work was supported by the Swiss Federal Office of Justice with two grants for the MAZ. and JAEL study awarded to MS. The neurobiological add-on study in which blood samples were drawn and biomarker analyzes were conducted was supported by the Gertrude Thalmann Foundation of the University Psychiatric Hospitals Basel, Switzerland (PI MS and DB). Furthermore, this work is currently supported by DFG/SNSF Lead Agency Grant (DFG: CL 878/2-1; SNSF: 204033; PI MS, VC, PK). DB was supported by a research grant from the Dr. Botond Berde-Fonds of the “Freie akademische Gesellschaft (FAG)” Basel and by the Research Fund for Excellent Junior Researcher of the University of Basel. KN was supported by US Department of Veterans Affairs Office of Research and Development (IK2CX002627).

## Declaration of competing interest

The authors declare no conflicts of interest.

## Data Availability

Data will be made available on request.
