# Supplementary material for: Associations of lifetime stressors and health behaviors with inflammation in young adults previously placed in youth residential care
Source: Brain Behav Immun Health. 2025 Sep 10;49:101098. doi: 10.1016/j.bbih.2025.101098 (PMC12629739; doi:10.1016/j.bbih.2025.101098)
Supplement: Multimedia component 1 [file mmc1.docx]

**Supplementary Material**

**Associations of lifetime stressors and health behaviors with inflammation in young adults previously placed in youth residential care**

**Supplementary Figure 1.** Flow Chart of Study Participants through the study baseline (MAZ. study), follow-up study (JAEL study) with final sample sizes included in data analyses.

Reason for drop-out (*n*=5):

- Not sufficient plasma samples available for inflammation assays (*n*=5)

Reason for drop-out (*n*=54):

- Afraid of needles (*n*=4)
- Digital interviews (*n*=8)
- Weekend Assessments/ lab not open (*n*=7)
- Did not want to (*n*=13)
- Other (*n*=22)

**Biomarker Sample (t2)**

Final Analytical Sample (*N*=126)

Reason for drop-out (*n*=81):

- Did not provide informed consent for follow-up (*n*=81)

**Follow-Up (JAEL, t2)**

**Baseline (MAZ., t1)**

Reason for drop-out (*n*=46):

- Unable to schedule a meeting and motivate participants to come to the in-person interviews (*n*=46)

Participants with blood sampling (*N*=131)

Reason for drop-out (*n*=280):

- Refused to participate (n=99)
- Oral agreement, but never participated (*n*=44)
- Could not be reached (*n*=129)
- Were deceased (*n*=8)

Included in the follow-up JAEL study (*N*=231)

Participants with consent for follow-up

(*N*=511)

Included in the baseline MAZ. study (*N*=592)

Face-to-Face Interviews of JAEL (*N*=185)

| **Supplementary Table 1.** Comparison included cases (*N*=126) versus non-included participants (*N*=466) at baseline (MAZ.) regarding psychosocial characteristics and mental health burden | | | | |
| --- | --- | --- | --- | --- |
|  | Included | Non-included | Test-Statistic | p-value |
|  | M (SD) or N (%) | M (SD) or N (%) |  |  |
| Age | 16.33 (2.61) | 16.24 (2.57) | t(157.60) = -0.32 | 0.750 |
| Sex (Female) | 39 (31%) | 155 (32.9%) | *χ^2^=0.096, df=1* | 0.757 |
| Nationality (Swiss) | 110 (90.9%) | 391 (83%) | *χ^2^=4.025, df=1* | **0.045*** |
| Reason for placement (t1) |  |  | *χ^2^=0.668, df=2* | 0.716 |
| Civil | 62 (52.1%) | 250 (54.1%) |  |  |
| Criminal | 29 (24.4%) | 119 (25.8%) |  |  |
| Other | 28 (23.5%) | 93 (20.1%) |  |  |
| Any Personality Disorder^1^ | 20 (21.7%) | 88 (25.4%) | *χ^2^=0.337, df=1* | 0.561 |
| Any Mental Disorder^2^ | 72 (73.5%) | 267 (69.9%) | *χ^2^=0.323, df=1* | 0.570 |
| Any Trauma^3^ | 47 (65.3%) | 177 (69.7%) | *χ^2^=0.323, df=1* | 0.570 |
| Self-rep. Psychopathology^4^ |  |  |  |  |
| Total (T-values) | 60.06 (11.92) | 61.31 (9.9) | t(140.79) = 0.99 | 0.325 |
| Internalizing (T-values) | 57.1 (12.4) | 57.9 (10.5) | t(142.41) = 0.61 | 0.542 |
| Externalizing (T-values) | 59.8 (11.44) | 61.34 (10.25) | t(147.40) = 1.25 | 0.213 |
| Psychopathic Traits^5^ | 112.63 (23.8) | 112.39 (22.31) | t(153.58) = -0.09 | 0.927 |
| Non-verbal reasoning (IQ)^6^ | 95.49 (14.63) | 95.94 (14.44) | t(129.21) = 0.25 | 0.801 |
| **Notes.** N=number of participants; IQ = intelligence quotient; total N of the tests performed vary between 326-592.  ^1^ assessed with the semi-structured clinical interview SCID-II [1,2]  ^2^ assessed with the semi-structured clinical interview Kiddie-SADS [2,3]  ^3^ assessed with the ‘Essen Trauma-Inventory (ETI-KJ)‘ [4]  ^4^ Dimensional psychopathology was self-reported by participants at baseline with the ‘Youth Self Report (YSR)‘ and the ‚Young Adult Self Report (YASR)‘ of the Achenbach scales, t-values reported [5,6]  ^5^ Youth Psychopathic Traits Inventory (YPI) sum score [7,8]  ^6^ assessed with the Standard Progressive Matrices of Raven or the Culture Fair Intelligence Test [9] | | | | |

| **Supplementary Table 2.** Comparison of included cases (N=126) versus non-included MAZ. participants (N=466) regarding follow-up data of criminal record data from the Swiss Federal Bureau of Statistics (BFS) up until the year 2017 | | | | |
| --- | --- | --- | --- | --- |
|  | Included, N (%) | Non-included, N (%) | Test-Statistic | p-value |
| Before the age of 18 |  |  |  |  |
| Any offense | 57 (47.1%) | 200 (42.5%) | χ^2^=0.667, df=1 | 0.414 |
| Violent offense | 20 (16.5%) | 72 (15.3%) | χ^2^=0.038, df=1 | 0.845 |
| Prison sentence | 9 (7.4%) | 23 (4.9%) | χ^2^=0.780, df=1 | 0.780 |
| After the age of 18 |  |  |  |  |
| Any offense | 40 (33.1%) | 156 (33.1%) | χ^2^=0.000, df=1 | 1.000 |
| Violent offense | 14 (11.6%) | 47 (10%) | χ^2^=0.120, df=1 | 0.729 |
| Prison sentence | 16 (13.2%) | 56 (11.9%) | χ^2^=0.060, df=1 | 0.807 |
| **Notes.** N=number of participants. Any Offense includes delicts classified as “Vergehen” or “Verbrechen” in Swiss criminal law. The known data of the delict was used and not of the verdict for age classification. More information on the data from the Swiss federal office of Statistics are reported elsewhere [8]. | | | | |

| **Supplementary Table 3**. Exploratory correlation matrix of all study variables of interest | | | | | | | | | | | | | | | |
| --- | --- | --- | --- | --- | --- | --- | --- | --- | --- | --- | --- | --- | --- | --- | --- |
| **Measure** | 2.  SLEs | 3.  Nicotine | 4.  Cannabis | 5.  Alcohol | 6.  Physical Activity | 7.  Nutrition | 8.  Sleep | 9.  CRP | 10.  IL-6 | 11.  TNF-α | 12.  IL-10 | 13.  IL-1ra | 14.  Age | 15.  Sex (male) | 16.  BMI |
| 1. ELS | **0.33^***^** | 0.11 | 0.17 | -0.07 | 0.02 | -0.02 | **-0.24^**^** | 0.13 | 0.03 | 0.01 | 0.01 | **0.19^*^** | 0.00 | **0.39^***^** | 0.07 |
| 2. SLEs | -- | 0.05 | 0.17 | -0.05 | -0.05 | -0.01 | **-0.24^**^** | 0.05 | 0.12 | 0.00 | 0.04 | 0.06 | 0.15 | -0.10 | 0.02 |
| 3. Nicotine |  | -- | **0.19^**^** | 0.17 | **-0.28^**^** | **-0.19^*^** | -0.07 | **0.30^***^** | **0.36^***^** | 0.05 | -0.03 | 0.16 | 0.17 | 0.01 | 0.00 |
| 4. Cannabis |  |  | -- | 0.17 | -0.17 | 0.04 | 0.01 | -0.11 | 0.09 | 0.02 | -0.10 | -0.04 | -0.05 | -0.05 | -0.20 |
| 5. Alcohol |  |  |  | -- | 0.12 | 0.14 | 0.07 | 0.01 | -0.03 | -0.03 | -0.16 | -0.13 | 0.07 | **0.18^*^** | 0.09 |
| 6. Physical Activity |  |  |  |  | -- | **0.27^**^** | 0.03 | -0.02 | **-0.18^*^** | 0.01 | 0.01 | -0.09 | -0.05 | -0.04 | 0.19 |
| 7. Nutrition |  |  |  |  |  | -- | 0.01 | -0.17 | **-0.20^*^** | -0.10 | -0.07 | 0.02 | -0.03 | -0.11 | 0.08 |
| 8. Sleep |  |  |  |  |  |  | -- | -0.03 | -0.16 | 0.02 | -0.10 | **-0.21^*^** | 0.06 | **0.24^**^** | -0.06 |
| 9. CRP |  |  |  |  |  |  |  | -- | **0.64^***^** | 0.09 | **0.20^*^** | **0.45^***^** | **0.27^**^** | 0.09 | **0.42^***^** |
| 10. IL-6 |  |  |  |  |  |  |  |  | -- | 0.08 | 0.13 | **0.46^***^** | **0.24^**^** | 0.14 | **0.22^*^** |
| 11. TNF-α |  |  |  |  |  |  |  |  |  | -- | **0.30^***^** | **0.31^***^** | -0.05 | 0.08 | 0.01 |
| 12. IL-10 |  |  |  |  |  |  |  |  |  |  | -- | 0.06 | -0.04 | 0.03 | 0.05 |
| 13. IL-1ra |  |  |  |  |  |  |  |  |  |  |  | -- | 0.08 | -0.10 | **0.38^***^** |
| 14. Age |  |  |  |  |  |  |  |  |  |  |  |  | -- | 0.01 | 0.10 |
| 15. Sex (male) |  |  |  |  |  |  |  |  |  |  |  |  |  | -- | 0.02 |
| 16. BMI |  |  |  |  |  |  |  |  |  |  |  |  |  |  | -- |
| **Notes.** Spearman correlation coefficients and corresponding significance tests. Inflammatory markers are log-transformed and standardized (except TNF-α, only standardized). ^***^ p<.001, ^**^ p<.01, ^*^ p<.05 | | | | | | | | | | | | | | | |

| **Supplementary Table 4.** Descriptive statistics on inflammatory markers for raw values and standardized and log-transformed values | | | | | | | | |
| --- | --- | --- | --- | --- | --- | --- | --- | --- |
| **Variable** | **Mean** | **SD** | **Median** | **MAD** | **Min** | **Max** | **Skewness** | **Kurtosis** |
| CRP (mg/L) | 4.52 | 10.27 | 1.73 | 1.67 | 0.18 | 77.29 | 5.00 | 27.31 |
| IL-6 (pg/mL) | 1.84 | 1.78 | 1.33 | 0.92 | 0.05 | 14.24 | 3.65 | 20.06 |
| TNF-α (pg/mL) | 0.73 | 0.31 | 0.66 | 0.27 | 0.02 | 1.70 | 0.83 | 0.94 |
| IL-10 (pg/mL) | 1.04 | 1.15 | 0.73 | 0.80 | 0.09 | 9.32 | 3.60 | 21.39 |
| IL-1ra (pg/mL) | 280.93 | 218.96 | 220.43 | 110.57 | 95.74 | 1795.95 | 3.99 | 21.93 |

| **Supplementary Table 5.** Associations of ELS and SLEs with inflammatory markers | | | | | | | | | | |
| --- | --- | --- | --- | --- | --- | --- | --- | --- | --- | --- |
|  | **CRP** | | **IL-6** | | **TNF-α** | | **IL-10** | | **IL-1ra** | |
| **Parameter** | β | 95%CI | β | 95%CI | β | 95%CI | β | 95%CI | β | 95%CI |
| Age | **0.18** | **0.02, 0.34^*^** | **0.24** | **0.07, 0.41^**^** | -0.05 | -0.23, 0.13 | -0.08 | -0.27, 0.10 | 0.00 | -0.17, 0.17 |
| Sex (male) | 0.36 | -0.03, 0.75 | 0.23 | -0.17, 0.63 | 0.30 | -0.12, 0.72 | -0.08 | -0.50, 0.34 | -0.01 | -0.41, 0.39 |
| BMI | **0.36** | **0.17, 0.54^***^** | **0.23** | **0.03, 0.42^*^** | 0.03 | -0.18, 0.23 | 0.07 | -0.13, 0.26 | **0.41** | **0.23, 0.59^***^** |
| ELS | 0.12 | -0.08, 0.31 | -0.01 | -0.21, 0.19 | 0.08 | -0.13, 0.30 | -0.06 | -0.27, 0.15 | 0.12 | -0.08, 0.31 |
| SLEs | -0.01 | -0.19, 0.17 | 0.06 | -0.13, 0.24 | 0.00 | -0.20, 0.19 | 0.09 | -0.11, 0.28 | -0.02 | -0.20, 0.16 |
| Model R^2^ | 0.21 | | 0.14 | | 0.02 | | 0.02 | | 0.20 | |
|  |  |  |  |  |  |  |  |  |  |  |
| ELS*SLEs | -0.01 | -0.17, 0.15 | 0.02 | -0.14, 0.18 | -0.01 | -0.19, 0.16 | -0.05 | -0.22, 0.12 | 0.01 | -0.147, 0.174 |
| **Notes.** Pooled estimates of multiple regression models across all imputed datasets as displayed in Figure 1. Different inflammatory markers are predicted by early life stressors (ELS), stressful life events (SLEs), age, sex, and BMI. Additionally, ELS*SLEs interactions are tested in additional models, from these models only the interactions are reported. All dimensional variables are standardized, all binary variables are coded using zero and one. Error bars represent pooled 95%-Confidence Intervals. ^***^ *p<.001*, ^**^ p<.01, ^*^ *p<.05* | | | | | | | | | | |

| **Supplementary Table 6.** Associations of risky and protective health behaviors with inflammatory markers | | | | | | | | | | |
| --- | --- | --- | --- | --- | --- | --- | --- | --- | --- | --- |
|  | **CRP** | | **IL-6** | | **TNF-α** | | **IL-10** | | **IL-1ra** | |
| **Parameter** | β | 95%CI | β | 95%CI | β | 95%CI | β | 95%CI | β | 95%CI |
| Age | 0.14 | -0.02, 0.30 | **0.21** | **0.05, 0.36^*^** | -0.06 | -0.25, 0.13 | -0.06 | -0.25, 0.12 | -0.01 | -0.17, 0.16 |
| Sex (male) | 0.27 | -0.10, 0.64 | 0.31 | -0.05, 0.67 | 0.25 | -0.17, 0.66 | 0.02 | -0.40, 0.43 | 0.05 | -0.33, 0.43 |
| BMI | **0.36** | **0.18, 0.54^***^** | **0.26** | **0.08, 0.45^**^** | 0.03 | -0.18, 0.24 | 0.05 | -0.15, 0.25 | **0.43** | **0.24, 0.61^***^** |
| Nicotine | **0.47** | **0.12, 0.82^**^** | **0.42** | **0.08, 0.77^*^** | 0.10 | -0.30, 0.50 | -0.08 | -0.48, 0.33 | 0.24 | -0.12, 0.60 |
| Cannabis | -0.11 | -0.52, 0.30 | 0.16 | -0.24, 0.57 | -0.01 | -0.48, 0.47 | -0.16 | -0.64, 0.31 | -0.05 | -0.47, 0.37 |
| Alcohol | -0.05 | -0.42, 0.32 | -0.09 | -0.46, 0.28 | -0.02 | -0.44, 0.41 | -0.23 | -0.65, 0.20 | -0.34 | -0.72, 0.04 |
| Physical Activity | -0.03 | -0.21, 0.15 | **-0.23** | **-0.41, -0.04^*^** | 0.01 | -0.19, 0.21 | -0.01 | -0.23, 0.21 | -0.13 | -0.33, 0.07 |
| Nutrition | -0.09 | -0.27, 0.09 | -0.03 | -0.21, 0.14 | -0.09 | -0.29, 0.11 | 0.00 | -0.22, 0.21 | 0.12 | -0.07, 0.32 |
| Sleep | -0.05 | -0.22, 0.11 | -0.16 | -0.32, 0.00 | -0.06 | -0.25, 0.12 | -0.07 | -0.26, 0.12 | -0.14 | -0.31, 0.03 |
| Model R^2^ | 0.29 | | 0.32 | | 0.04 | | 0.04 | | 0.27 | |
| **Notes.** Pooled estimates of multiple regression models across all imputed datasets as displayed in Figure 2. Different inflammatory markers are predicted by individual risky and protective health behaviors, age, sex, and BMI. All dimensional variables are standardized, all binary variables are coded using zero and one. Error bars represent pooled 95%-Confidence Intervals. ^***^ *p<.001*, ^**^ p<.01, ^*^ *p<.05* | | | | | | | | | | |

| **Supplementary Table 7.** Associations of ELS, SLEs, and risky and protective health behaviors with inflammatory markers | | | | | | | | | | |
| --- | --- | --- | --- | --- | --- | --- | --- | --- | --- | --- |
|  | **CRP** | | **IL-6** | | **TNF-α** | | **IL-10** | | **IL-1ra** | |
| **Parameter** | β | 95%CI | β | 95%CI | β | 95%CI | β | 95%CI | β | 95%CI |
| Age | 0.14 | -0.02, 0.31 | **0.21** | **0.05, 0.37^*^** | -0.06 | -0.25, 0.13 | -0.07 | -0.26, 0.12 | 0.00 | -0.17, 0.16 |
| Sex (male) | 0.34 | -0.06, 0.74 | 0.26 | -0.12, 0.65 | 0.30 | -0.15, 0.74 | -0.01 | -0.46, 0.43 | 0.12 | -0.29, 0.53 |
| BMI | **0.35** | **0.17, 0.54^***^** | **0.27** | **0.08, 0.46^**^** | 0.02 | -0.19, 0.24 | 0.06 | -0.15, 0.26 | **0.42** | **0.23, 0.60^***^** |
| ELS | 0.10 | -0.10, 0.30 | -0.06 | -0.26, 0.13 | 0.07 | -0.15, 0.29 | -0.05 | -0.27, 0.17 | 0.11 | -0.09, 0.31 |
| SLEs | -0.03 | -0.21, 0.16 | -0.03 | -0.21, 0.15 | -0.02 | -0.22, 0.19 | 0.07 | -0.13, 0.28 | -0.08 | -0.26, 0.10 |
| Nicotine | **0.46** | **0.11, 0.81^*^** | **0.43** | **0.08, 0.77^*^** | 0.10 | -0.31, 0.50 | -0.07 | -0.48, 0.33 | 0.24 | -0.12, 0.59 |
| Cannabis | -0.15 | -0.58, 0.28 | 0.20 | -0.22, 0.63 | -0.03 | -0.52, 0.46 | -0.17 | -0.66, 0.32 | -0.07 | -0.51, 0.36 |
| Alcohol | -0.04 | -0.42, 0.33 | -0.09 | -0.46, 0.28 | -0.02 | -0.44, 0.41 | -0.22 | -0.65, 0.21 | -0.34 | -0.73, 0.04 |
| Physical Activity | -0.04 | -0.22, 0.15 | **-0.22** | **-0.41, -0.04^*^** | 0.01 | -0.20, 0.21 | -0.01 | -0.23, 0.21 | -0.14 | -0.34, 0.06 |
| Nutrition | -0.08 | -0.26, 0.10 | -0.04 | -0.21, 0.14 | -0.08 | -0.29, 0.12 | -0.01 | -0.23, 0.21 | 0.13 | -0.07, 0.33 |
| Sleep | -0.04 | -0.22, 0.14 | **-0.18** | **-0.36, -0.01^*^** | -0.05 | -0.26, 0.15 | -0.06 | -0.26, 0.15 | -0.14 | -0.33, 0.04 |
| Model R^2^ | 0.29 | | 0.32 | | 0.04 | | 0.04 | | 0.27 | |
| **Notes.** Pooled estimates of multiple regression models across all imputed datasets as displayed in Figure 3. Different inflammatory markers are predicted by ELS, SLEs, individual risky and protective health behaviors, age, sex, and BMI. All dimensional variables are standardized, all binary variables are coded using zero and one. Error bars represent pooled 95%-Confidence Intervals. ^***^ *p<.001*, ^**^ p<.01, ^*^ *p<.05* | | | | | | | | | | |

1. d’Huart D, Steppan M, Seker S, Bürgin D, Boonmann C, Birkhölzer M, et al. Prevalence and 10-Year Stability of Personality Disorders From Adolescence to Young Adulthood in a High-Risk Sample. Front Psychiatry. 2022;13: 840678. doi:10.3389/fpsyt.2022.840678

2. Dölitzsch C, Fegert JM, Künster A, Kölch M, Schmeck K, Schmid M. Mehrfachdiagnosen bei Schweizer Heimjugendlichen. Kindheit und Entwicklung. 2014;23: 140–150. doi:10.1026/0942-5403/a000140

3. Seker S, Boonmann C, Gerger H, Jäggi L, d’Huart D, Schmeck K, et al. Mental disorders among adults formerly in out-of-home care: a systematic review and meta-analysis of longitudinal studies. Eur Child Adolesc Psychiatry. 2022;31: 1963–1982. doi:10.1007/s00787-021-01828-0

4. Fischer S, Dölitzsch C, Schmeck K, Fegert JM, Schmid M. Interpersonal trauma and associated psychopathology in girls and boys living in residential care. Children and Youth Services Review. 2016;67: 203–211. doi:10.1016/j.childyouth.2016.06.013

5. Dölitzsch C, Kölch M, Fegert JM, Schmeck K, Schmid M. Ability of the Child Behavior Checklist-Dysregulation Profile and the Youth Self Report-Dysregulation Profile to identify serious psychopathology and association with correlated problems in high-risk children and adolescents. J Affect Disord. 2016;205: 327–334. doi:10.1016/j.jad.2016.08.010

6. Seker S, Bürgin D, d’Huart D, Schmid M, Schmeck K, Jenkel N, et al. Der Verlauf von psychischen Problemen bei fremdplatzierten Kindern und Jugendlichen bis in deren Erwachsenenalter. Kindheit und Entwicklung. 2022 [cited 5 Mar 2025]. Available: https://econtent.hogrefe.com/doi/10.1026/0942-5403/a000365

7. Hachtel H, Jenkel N, Schmeck K, Graf M, Fegert JM, Schmid M, et al. Stability of self-reported psychopathic traits in at-risk adolescents in youth welfare and juvenile justice institutions. Child and Adolescent Psychiatry and Mental Health. 2022;16: 55. doi:10.1186/s13034-022-00487-6

8. Jäggi L, Schmid M, Bürgin D, Saladin N, Grob A, Boonmann C. Shared residential placement for child welfare and juvenile justice youth: current treatment needs and risk of adult criminal conviction. Child Adolesc Psychiatry Ment Health. 2021;15: 2. doi:10.1186/s13034-020-00355-1

9. Habersaat S, Ramain J, Mantzouranis G, Palix J, Boonmann C, Fegert JM, et al. Substance-use disorders, personality traits, and sex differences in institutionalized adolescents. Am J Drug Alcohol Abuse. 2018;44: 686–694. doi:10.1080/00952990.2018.1491587
